# Beyond the golden hour: prehospital delay and in-hospital stroke mortality in an elderly cohort reaching a national emergency-medicine center in Kazakhstan

**DOI:** 10.3389/fneur.2026.1916069

**Published:** 2026-08-18

**Authors:** Aigul A. Abduldayeva, Aliya M. Omarbek, Gulnur N. Doszhanova, Yerzhan B. Adilbekov, Nikolay A. Safonov, Saule A. Iskakova

**Affiliations:** 1 Laboratory of Health, Research Institute of Preventive Medicine, NCJSC “Astana Medical University”, Astana, Kazakhstan; 2 National Coordination Center for Emergency Medicine, Astana, Kazakhstan

**Keywords:** cardiac arrhythmia, elderly, emergency medical services, in-hospital mortality, Kazakhstan, prehospital delay, reperfusion, stroke

## Abstract

Stroke remains a leading cause of death and disability worldwide, and the “time-is-brain” paradigm frames prehospital speed as the principal modifiable determinant of outcome. Data from Kazakhstan, a country of about 2.72 million km^2^ with a population density of roughly 7 inhabitants per km^2^, are scarce, and it is unclear whether time metrics retain their prognostic role where most patients present outside reperfusion windows. We retrospectively analyzed 149 elderly patients (aged 57–75 years) with neuroimaging-confirmed acute stroke admitted to the National Coordination Center for Emergency Medicine (Astana, Kazakhstan) in 2025–2026; transient ischaemic attacks were excluded. Prehospital time, transport mode, reperfusion, neurological severity (NIHSS), comorbidity and in-hospital mortality were assessed using Spearman correlation, ANOVA and logistic regression. Ischemic stroke predominated (122/149; 81.9%). The median onset-to-door time was 9.5 h (IQR 3.0–48.3 h; range 25 min to 264 h), and only 36.5% arrived within the 4.5-h thrombolysis window; delays were longest in hemorrhagic stroke (median 24.8 h). Reperfusion therapy reached 24.6% of ischemic-stroke patients (thrombolysis 9.0%, thrombectomy 15.6%), whereas 67.2% were managed conservatively. Despite these delays, in-hospital mortality was low (6.8%). Onset-to-door time predicted neither in-hospital death (adjusted OR 1.00 per hour; *p* = 0.99) nor discharge functional status (modified Rankin Scale *r* = −0.013, *p* = 0.87); in a pre-specified Firth penalized model the predictors of death were baseline NIHSS (penalized OR 1.22 per point; 95% profile-likelihood CI 1.12–1.37; *p* < 0.001) and cardiac arrhythmia (penalized OR 5.51; 95% CI 1.07–29.71; *p* = 0.041). The crude mortality contrast (29.4% vs. 3.8%) is descriptive and hypothesis-generating. Baseline NIHSS was inversely correlated with onset-to-door time (*r* = −0.193; *p* = 0.019), indicating that later-presenting patients were less severely affected. These findings apply specifically to elderly patients who survived to reach the center and are not causal: the severity–time and time–mortality associations reflect who reaches the center. Within this hospital-admitted cohort, in-hospital mortality appears governed by neurological severity and cardiac comorbidity rather than transport time, supporting risk-based triage, arrhythmia detection, regionalized routing and air-ambulance capacity alongside efforts to compress door times.

## 1 Introduction

Stroke is among the leading causes of death and acquired disability worldwide, and its absolute burden continues to rise with population aging; most deaths and disability-adjusted life-years now occur in low- and middle-income countries (1–3). The therapeutic logic of acute stroke care rests on the “time-is-brain” principle: the benefit of intravenous thrombolysis and endovascular thrombectomy falls steeply with every minute of delay, and modern systems are engineered to shorten the interval from symptom onset to reperfusion (4–7). Prehospital emergency medical services (EMS) are the entry point to this system, responsible for recognition, triage, prenotification and routing (8, 9).

Almost all of the evidence underpinning this paradigm comes from high-income, urbanized health systems with dense networks of stroke centers. Kazakhstan presents an instructive contrast. It is the ninth-largest country in the world by area (approximately 2.72 million km^2^) but has a population of about 20 million, giving a population density of roughly 7 inhabitants per km^2^ - among the lowest worldwide. The population is highly unevenly distributed, concentrated in a small number of urban agglomerations, while vast central, western and northern territories are sparsely inhabited; reperfusion-capable centers are correspondingly concentrated in major cities, so that patients in remote districts depend on long ground transfers or on air ambulance. Under such conditions the preconditions for time-critical care are often absent: long transport distances, uneven access to reperfusion-capable hospitals and limited standardization of prehospital triage (10, 11). Central Asia, and Kazakhstan in particular, is almost absent from the prehospital stroke literature (10, 12), even though national statistics document a substantial and rising stroke burden (13).

It is therefore unknown whether, in such a setting, classical time metrics remain the dominant determinants of outcome, or whether other factors come to the fore once most patients have already missed the reperfusion window. We addressed this question in a real-world cohort of elderly stroke patients managed through the national emergency-medicine coordination system of Kazakhstan. Rather than asking only how fast patients arrive, we asked whether prehospital time actually predicts in-hospital mortality in this context and, if not, what does. We hypothesized that, in a system where the reperfusion window is routinely exceeded, neurological severity and cardiac comorbidity - rather than transport logistics - would govern in-hospital mortality.

## 2 Materials and methods

### 2.1 Study design and setting

We performed a retrospective analytical study at the National Coordination Center for Emergency Medicine and the Laboratory of Health, Research Institute of Preventive Medicine, NCJSC “Astana Medical University.” The Center is a national referral and air-ambulance coordination facility that organizes emergency stroke care across a large, low-population-density territory, making it a representative window onto prehospital stroke care in this setting. Importantly, patients reaching the Center are those judged fit to survive inter-hospital transfer, whereas the most haemodynamically unstable patients may be managed in peripheral hospitals without transfer; this is considered in the interpretation of our findings.

### 2.2 Participants

Consecutive elderly patients admitted with acute stroke during 2025–2026 were screened. Inclusion criteria were a neuroimaging-confirmed diagnosis of stroke, admission to the Center, and complete medical documentation. Exclusion criteria were incomplete clinical data, transient ischaemic attack (TIA), and repeat admissions of the same patient (only the index event was retained). Because TIA was an explicit exclusion criterion, the cohort contains no TIAs: all 122 ischemic cases were completed infarcts confirmed by computed tomography or magnetic resonance imaging. The final sample comprised 149 patients, classified by stroke type into ischemic (*n* = 122) and hemorrhagic (*n* = 27).

### 2.3 Variables and definitions

Data were abstracted from inpatient records, emergency-department documentation, neuroimaging (CT/MRI), post-mortem clinical summaries for fatal cases, and EMS records. We analyzed clinico-demographic variables (age, sex, type 2 diabetes, cardiac arrhythmia, hypertension grade, lipid profile); clinical variables (stroke severity by the National Institutes of Health Stroke Scale [NIHSS] at admission and discharge, functional status by the modified Rankin Scale and Barthel Index at discharge, intensive-care length of stay); organizational variables (onset-to-door time in minutes; mode of transport: EMS ambulance, air ambulance, or self-referral); the acute treatment received (conservative management, diagnostic angiography only, intravenous thrombolysis, or thrombectomy/selective intra-arterial therapy); and the primary endpoint, in-hospital mortality (survival to discharge vs. death). Discharge functional status was analyzed as a secondary endpoint. Prehospital timeliness was additionally categorized against clinically meaningful thresholds (<4.5 h, <6 h, <24 h).

### 2.4 Statistical analysis

Analyses were performed in IBM SPSS Statistics v.31. Because onset-to-door time was strongly right-skewed, it is summarized as median, interquartile range (IQR) and full range in addition to mean ± standard error. Associations between quantitative variables used Pearson or Spearman correlation as appropriate; categorical variables were compared by the χ^2^ test and groups by one-way ANOVA. The primary analysis of in-hospital death was a pre-specified parsimonious model containing only two *a priori* predictors - baseline NIHSS and cardiac arrhythmia - fitted by Firth penalized logistic regression, with penalized odds ratios (OR) and profile-likelihood 95% confidence intervals (CI); this approach was chosen *a priori* because only 10 deaths occurred. A full six-covariate maximum-likelihood model (adding age, intensive-care stay, onset-to-door time in hours, and stroke type) is reported as an exploratory sensitivity analysis; because onset-to-door time was strongly right-skewed it was modeled per hour, with a log10-hour scale examined as a check. A two-sided *p* < 0.05 was considered significant.

### 2.5 Ethics statement

The study was approved by the Local Bioethics Committee of NCJSC “Astana Medical University” (protocol No. 17, 9 December 2025). Owing to the retrospective design and the use of anonymized data, the requirement for individual informed consent was waived.

## 3 Results

### 3.1 Cohort characteristics

Of 149 elderly patients, ischemic stroke accounted for 122 (81.9%) and hemorrhagic stroke for 27 (18.1%); there were no TIAs, which were excluded by design. Ages ranged from 57 to 75 years (mean 66.8; median 67): 60–75 years in ischemic stroke (mean 67.1 ± 0.4) and 57–74 years in hemorrhagic stroke (mean 65.4 ± 0.8). Severe hypertension was present in 68.5% and type 2 diabetes in 26.8%; cardiac arrhythmia was documented in 17 patients (11.4%). Hemorrhagic stroke was associated with a longer intensive-care stay (12.7 ± 1.5 vs. 8.3 ± 0.4 days; *p* < 0.001), while type 2 diabetes was numerically more frequent in ischemic stroke (30.6% vs. 11.1%; χ^2^ *p* = 0.069). Male sex was more common in ischemic than hemorrhagic stroke (59.0% vs. 29.6%; *p* = 0.011). Baseline characteristics are shown in Table 1.

**Table 1:** Baseline and clinical characteristics by stroke type (*n* = 149).

Characteristic	Ischemic (*n* = 122)	Hemorrhagic (*n* = 27)	*p*
Age, years (M ± SE)	67.1 ± 0.4	65.4 ± 0.8	ns
Age range, years (min–max)	60–75	57–74	—
Male sex, *n* (%)	72 (59.0)	8 (29.6)	0.011
Baseline NIHSS, points	7.3 ± 0.6	6.0 ± 1.4	ns
Cardiac arrhythmia, *n* (%)	16 (13.1)	1 (3.7)	0.290
Type 2 diabetes, *n* (%)	37 (30.6)	3 (11.1)	0.069
Severe hypertension, *n* (%)	82 (67.2)	20 (74.1)	0.642
ICU length of stay, days	8.3 ± 0.4	12.7 ± 1.5	<0.001
Total cholesterol, mmol/L	4.87 ± 0.12	4.86 ± 0.19	ns

M ± SE, mean ± standard error; NIHSS, National Institutes of Health Stroke Scale; ICU, intensive care unit; ns, not significant. Categorical variables were compared by the χ^2^ test and continuous variables by ANOVA/*t*-test.

### 3.2 Prehospital delay far exceeds reperfusion windows

**Figure 1:**
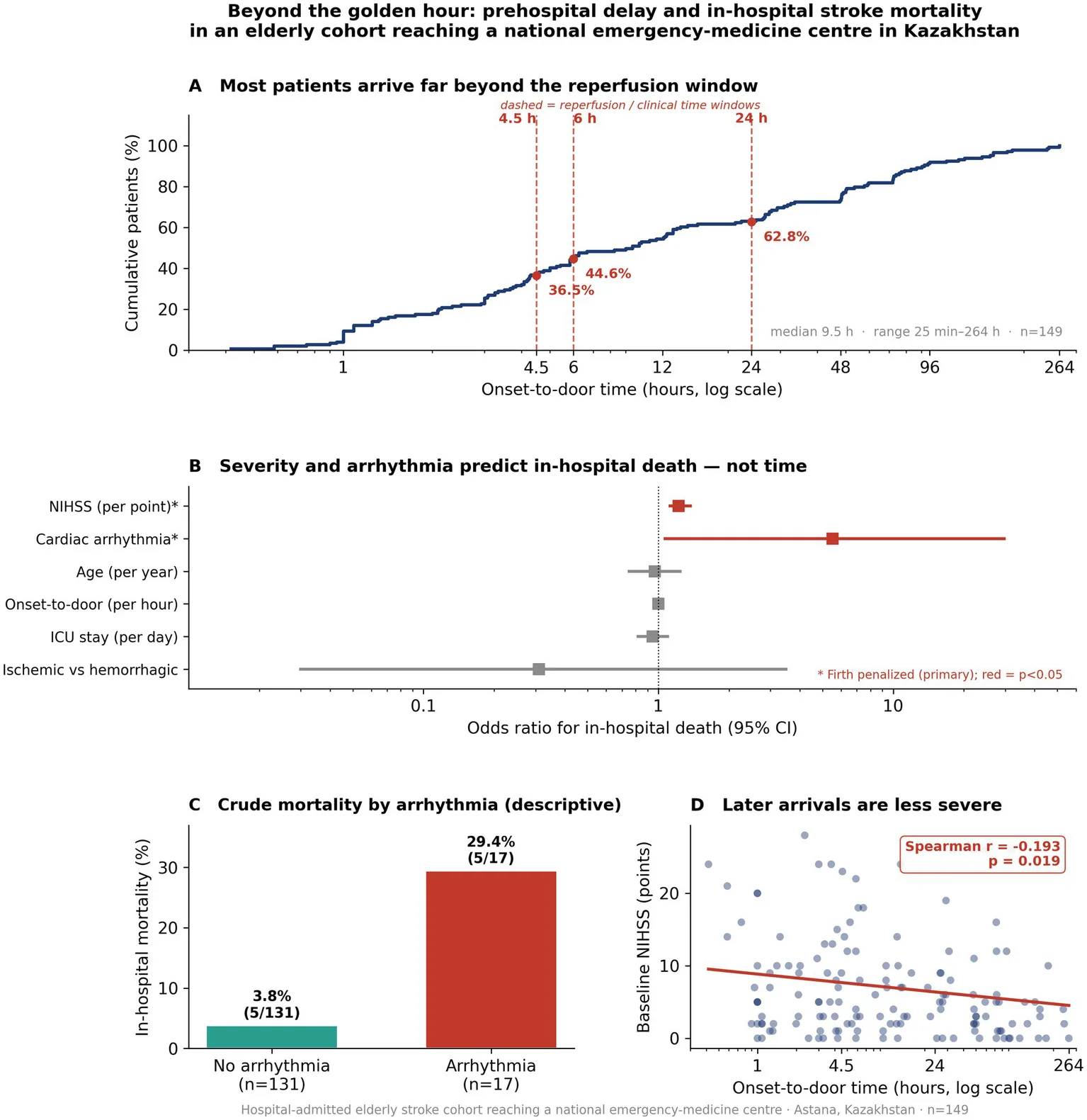
Beyond the golden hour. **(A)** Cumulative onset-to-door time against reperfusion/clinical time windows; only 36.5% arrive within 4.5 h (range 25 min–264 h). **(B)** Odds ratios for in-hospital death (onset-to-door time per hour); red indicates *p* < 0.05. **(C)** Crude in-hospital mortality by cardiac arrhythmia status (descriptive; 5 events per group). **(D)** Baseline NIHSS against onset-to-door time, showing that later-presenting patients were less severely affected (Spearman *r* = −0.193; *p* = 0.019). Elderly stroke cohort, National Coordination Center for Emergency Medicine, Astana, Kazakhstan (*n* = 149).

The defining feature of this cohort was the magnitude of prehospital delay. The median onset-to-door time was 9.5 h (IQR 3.0–48.3 h), ranging from 25 min to 264 h (11 days). Only 36.5% of patients reached the Center within the 4.5-h thrombolysis window; 44.6% within 6 h and 62.8% within 24 h (Figure 1A). Delay was markedly longer in hemorrhagic stroke (median 24.8 h) than in ischemic stroke (median 9.0 h). Most patients arrived by EMS ambulance (81.2%), with air ambulance accounting for 7.4% and self-referral for 11.4%; air-ambulance use differed markedly by stroke type (33.3% in hemorrhagic versus 1.6% in ischemic stroke), reflecting transfer of severe cases from remote regions.

Mode of transport correlated with neurological severity (NIHSS *r* ≈ −0.27 to −0.36; *p* < 0.001), indicating functioning but coarse severity-based prioritization. Importantly, baseline NIHSS was inversely correlated with onset-to-door time (Spearman *r* = −0.193; *p* = 0.019; *n* = 149): patients presenting late were on average less severely affected (median baseline NIHSS 5.0 in those arriving within 4.5 h versus 4.0 in those arriving at or beyond 24 h) (Figure 1D). Prehospital metrics are summarized in Table 2.

**Table 2:** Prehospital time, mode of transport and acute treatment.

Prehospital metric	Overall (*n* = 148)	By stroke type
Onset-to-door time, median (IQR), h	9.5 (3.0–48.3)	IS 9.0; HS 24.8
Onset-to-door time, range	25 min – 264 h	IS max 182 h; HS max 264 h
Arrived within 4.5 h, *n* (%)	54 (36.5)	IS 35.2; HS 44.4
Arrived within 6 h, *n* (%)	66 (44.6)	IS 45.1; HS 44.4
Arrived within 24 h, *n* (%)	93 (62.8)	IS 66.4; HS 48.1
Transport: EMS / air ambulance / self-referral, %	81.2/7.4/11.4	IS 85.2/1.6/13.1; HS 63.0/33.3/3.7
Intravenous thrombolysis, *n* (% of ischemic)	11 (9.0)	ischemic only
Thrombectomy / selective intra-arterial therapy, *n* (%)	19 (15.6)	ischemic only
Any reperfusion therapy, *n* (%)	30 (24.6)	ischemic only
Conservative management, *n* (%)	82 (67.2)	ischemic only
Diagnostic angiography only, *n* (%)	7 (5.7)	ischemic only

IS, ischemic stroke; HS, hemorrhagic stroke; IQR, interquartile range; EMS, emergency medical services. Treatment percentages refer to the 122 patients with ischemic stroke.

### 3.3 The reperfusion gap

Consistent with these delays, reperfusion therapy reached a minority of patients with ischemic stroke. Intravenous thrombolysis was administered to 11 patients (9.0%) and thrombectomy, selective intra-arterial thrombolysis or stenting to 19 (15.6%), giving a total reperfusion rate of 30/122 (24.6%); 82 patients (67.2%) were managed conservatively and 7 (5.7%) underwent diagnostic cerebral angiography only.

Among the 43 patients with ischemic stroke who arrived within 4.5 h, 17 received reperfusion (9 thrombolysis, 8 thrombectomy/intra-arterial therapy), 22 were managed conservatively and 4 underwent angiography only. In these in-window patients the decision to withhold thrombolysis was clinical rather than logistical: a low NIHSS score is not in itself an indication for thrombolysis, and treatment was decided on the clinical picture and on the severity and functional significance of the deficit. Patients managed conservatively within the window had mild strokes without functionally disabling deficits, so that the expected benefit did not outweigh the hemorrhagic risk, in line with guidance advising against routine thrombolysis in mild, non-disabling stroke (4). For descriptive illustration of this group, their median baseline NIHSS was 3.0 (IQR 2–7; 72.7% scoring ≤5) versus 9.0 (IQR 5–13) in those reperfused, and their discharge status was favorable (median modified Rankin Scale 2.0; median Barthel Index 82.5); we emphasize that this severity difference was not statistically significant (Mann–Whitney *p* = 0.111) and is presented as illustration rather than as a supported comparison. The decision to withhold thrombolysis was thus a clinical judgment based on the character and functional significance of the deficit, not on a formal statistical contrast. Notably, the NIHSS does not always capture the functional significance of a deficit: even a low score may be disabling when it involves, for example, paresis of the dominant hand, aphasia in a person whose occupation depends on speech, or loss of fine motor control; no such disabling deficit was present in the untreated patients. Individual contraindications were not systematically coded in the analytic dataset. Overall, the great majority of patients were managed outside the therapeutic window on which the time-is-brain model depends.

### 3.4 Determinants of in-hospital mortality

Despite the extreme prehospital delays and the low reperfusion rate, in-hospital mortality was low at 6.8% (10/148; one patient had a missing outcome). The strongest signal was cardiac: crude mortality was 29.4% (5/17) among patients with cardiac arrhythmia versus 3.8% (5/131) without - a crude risk ratio of about 7.7 based on five events per group, which we report descriptively and treat as hypothesis-generating (Figure 1C). In-hospital mortality correlated with NIHSS (*r* ≈ −0.33 to −0.37), the modified Rankin Scale (*r* = −0.391) and the Barthel Index (*r* = 0.435; all *p* < 0.001), and with cardiac arrhythmia (*r* = −0.325; *p* < 0.001).

In the pre-specified Firth penalized model, baseline NIHSS was associated with in-hospital death (penalized OR 1.22 per point; 95% profile-likelihood CI 1.12–1.37; *p* < 0.001), as was cardiac arrhythmia (penalized OR 5.51; 95% CI 1.07–29.71; *p* = 0.041); the arrhythmia interval, though excluding 1, remains wide and this estimate should be read cautiously given 10 events. In the exploratory six-covariate sensitivity model, onset-to-door time (OR 1.00 per hour; 95% CI 0.98–1.02; *p* = 0.99), age (OR 0.965; *p* = 0.778), intensive-care length of stay (OR 0.947; *p* = 0.466) and stroke type showed no association with mortality (Table 3; Figure 1B). The inverse severity–time correlation reported above and this null association between time and mortality are consequences of the referral/transport selection that determines which patients reach our Center, and must not be interpreted causally.

**Table 3:** **(A)** Descriptive in-hospital mortality by subgroup, **(B)** pre-specified primary model: Firth penalized logistic regression with the two a priori predictors, penalized odds ratios and profile-likelihood intervals, **(C)** exploratory six-covariate maximum-likelihood sensitivity model (onset-to-door time per hour).

A. In-hospital mortality by subgroup (descriptive)		
Subgroup	Deaths/*n*	Mortality, %
Overall	10/148	6.8
Cardiac arrhythmia – present	5/17	29.4
Cardiac arrhythmia – absent	5/131	3.8

B. Primary model: Firth penalized logistic regression (pre-specified; 10 events)			
Predictor	Penalized OR	95% profile-likelihood CI	*p*
Baseline NIHSS (per 1 point)	1.22	1.12–1.37	<0.001
Cardiac arrhythmia (present vs. absent)	5.51	1.07–29.71	0.041

C. Sensitivity: full six-covariate ML model (exploratory)			
Factor	OR	95% CI	*p*
Baseline NIHSS (per 1 point)	1.259	1.115–1.423	<0.001
Cardiac arrhythmia (present vs. absent)	10.3	1.13–90.9	0.039
Age (per year)	0.965	0.752–1.238	0.778
Onset-to-door time (per hour)	1.00	0.98–1.02	0.99
ICU length of stay (per day)	0.947	0.819–1.096	0.466
Ischemic vs. hemorrhagic stroke	0.31	0.03–3.50	0.340

The reference category for cardiac arrhythmia is its absence. OR, odds ratio; CI, confidence interval; NIHSS, National Institutes of Health Stroke Scale; ICU, intensive care unit.

In a secondary analysis of discharge functional status, onset-to-door time was associated neither with the modified Rankin Scale (Spearman *r* = −0.013; *p* = 0.87) nor with the Barthel Index (*r* = 0.058; *p* = 0.48), nor with NIHSS at discharge (*r* = −0.081; *p* = 0.33). In an exploratory analysis, non-survivors had somewhat higher total cholesterol and LDL than survivors (5.35/4.23 vs. 4.83/3.49 mmol/L), but with only 10 deaths this finding is hypothesis-generating.

Review of the post-mortem clinical summaries of all 10 fatal cases identified cerebral oedema with mass effect and herniation as the leading documented mechanism, recorded in 7 of 10 patients (in several cases explicitly as cerebral oedema with herniation into the foramen magnum accompanied by acute cardiovascular and respiratory failure). Pneumonia (aspiration, bronchial or hypostatic) was a major contributing complication in 5 of 10; acute cardiovascular and/or respiratory failure was the documented terminal event in 4 of 10, with suspected pulmonary embolism in 1. The fatal cases comprised 7 ischemic strokes (including large middle-cerebral-artery territory infarction, one with haemorrhagic transformation after thrombectomy, and brainstem infarction) and 3 intracerebral hemorrhages with intraventricular extension. Consistent with the regression results, a documented cardiac rhythm disorder - predominantly atrial fibrillation - was present in 5 of the 10 fatal cases.

## 4 Discussion

In this elderly stroke cohort from a large upper-middle-income country, prehospital reality diverged sharply from the assumptions of the dominant stroke-care paradigm. The median time from symptom onset to hospital arrival was 9.5 h - roughly an order of magnitude beyond the 4.5-h thrombolysis window and the “golden hour” that anchor high-income systems (4–7) - and almost two-thirds of patients arrived too late for standard reperfusion, so that only 24.6% received reperfusion therapy. Yet the central finding is not simply that delays are long, but that, in this cohort, onset-to-door time was not an independent determinant of in-hospital mortality, nor was it associated with discharge functional status. Instead, in-hospital mortality was associated with baseline neurological severity (NIHSS) and, more tentatively, with cardiac arrhythmia, which was documented in half of the fatal cases; with only 10 deaths the arrhythmia estimate is imprecise even after Firth penalization and is best regarded as hypothesis-generating. The male predominance in ischaemic stroke is consistent with reported sex differences in stroke presentation that are largely driven by age and severity (14).

Two mechanisms plausibly explain why time lost its prognostic weight here, and both temper any causal reading of this result. First, self-selection by severity: baseline NIHSS was inversely correlated with onset-to-door time, meaning that patients who presented very late were, on average, those with milder deficits who survived long enough to seek care, while severe strokes were recognized and transported faster (15). Second, and equally important, transport-related selection: our Center is a national referral and air-ambulance coordination facility, and patients transferred to it are by necessity those judged able to survive transfer, whereas the most critically unstable patients are frequently managed in peripheral hospitals without ever being transferred. This survivorship/referral bias plausibly contributes to the low in-hospital mortality (6.8%) observed despite extreme delays and to the absence of an independent time–mortality association. Our mortality estimate therefore applies to elderly patients who survived to reach the center (the hospital-admitted cohort), not to the national stroke population.

Accordingly, our findings should be read as a reframing rather than a rejection of time-is-brain. We do not claim that prehospital delay is unimportant for stroke outcome in general: our data cannot speak to 90-day disability, to outcomes among patients never transferred, or to the well-established time-dependence of reperfusion benefit (4–7). What our data do show is that, in a large-territory setting where reperfusion reaches only about a quarter of ischemic strokes, the marginal prognostic value of shaving minutes off an already out-of-window transport is small, and patient-level biology - stroke severity and cardioembolic burden - dominates in-hospital mortality. The strong arrhythmia signal is mechanistically coherent: atrial fibrillation and related rhythm disorders produce larger, more disabling cardioembolic infarcts and carry higher early mortality, and they also mark frailty and cardiac comorbidity in the elderly.

The reasons for the observed delays are structural as much as behavioral. The catchment area is vast: in a country of approximately 2.72 million km^2^ with a population density of about 7 inhabitants per km^2^ and a population concentrated in a few urban centers, ground transfer from remote districts can take many hours. Limited public awareness of stroke symptoms and of the need for an immediate emergency call contributes to care-seeking delay, as documented in comparable settings (15), as does uneven regional availability of emergency medical services (16, 17). The pattern of air-ambulance use in our cohort - 33.3% in hemorrhagic versus 1.6% in ischemic stroke - indicates that aeromedical transfer is currently reserved for the most severe or most remote cases.

These observations align with the small but growing literature on prehospital stroke care in low- and middle-income countries, which emphasizes distance, fragmented systems and limited reperfusion access (10, 11), with documented care-seeking delays (15) and EMS-related disparities elsewhere (16, 17). They extend that literature by quantifying, in a previously unrepresented Central Asian setting, both the magnitude of delay and its limited prognostic weight relative to comorbidity. For Kazakhstan, where stroke services are still maturing (12), the practical implication is a shift in emphasis: from the - currently unattainable - goal of universal door-time compression toward risk-based triage and system redesign. Concretely, this means standardized severity-based prehospital sorting (e.g., NIHSS-oriented and validated large-vessel-occlusion scales) (18, 19), systematic detection and flagging of cardiac arrhythmia at first contact, expansion and protocolization of air-ambulance use for remote regions, regionalized routing of severe and hemorrhagic strokes to appropriate centers, and hospital prenotification (8, 9, 20). Telemedicine-supported triage and mobile stroke units, where feasible, may further help bridge distance (5, 19). Prospective audit of why reperfusion is withheld in patients who do arrive within the window is a further priority.

Our findings should be read against the emerging Kazakhstani evidence base. Recent work from a tertiary stroke center in the Karaganda region reported a reperfusion rate of about 24% - closely matching ours - confirming that even well-organized centers in this setting treat only a minority of ischaemic strokes (21). National epidemiological analyses have documented the rising stroke burden and substantial regional disparities in prehospital timeliness across Kazakhstan (22, 23), but have focused on incidence, mortality and in-hospital care rather than on what determines outcome once the reperfusion window is missed. Our contribution is to show, in an elderly cohort, that in this circumstance prehospital time loses its independent prognostic weight for in-hospital death while neurological severity and cardiac arrhythmia dominate. This echoes earlier observations that prehospital delay has changed little over decades and that time-to-treatment does not uniformly translate into outcome (24). The arrhythmia signal we observed is itself contested - some cohorts identify atrial fibrillation as an independent predictor of in-hospital death (25), whereas propensity-matched analyses do not (26) - so, with few deaths, our data should be read as a hypothesis-generating data point from an under-represented region rather than as definitive.

### 4.1 Limitations

This was a single-center retrospective study, which limits causal inference and generalizability and is subject to documentation bias. Most importantly, the cohort is subject to transport-related selection: only patients considered able to survive inter-hospital transfer reach our Center, so our mortality estimate and the observed absence of a time–mortality association may not apply to patients who are never transferred. Our endpoint was in-hospital mortality; functional status was assessed at discharge rather than at 90 days, so we cannot exclude an effect of prehospital delay on longer-term disability. Onset times partly relied on patient or relative report, which is especially uncertain in elderly patients with cognitive impairment. Individual contraindications to reperfusion were not systematically coded in the analytic dataset, although the clinical rationale in patients arriving within the window -mild, non-disabling deficits - is supported by their NIHSS distribution and discharge status. Distance from the place of symptom onset or residence to the admitting hospital was not documented and could not be reconstructed, as the dataset contains no geographic identifiers; onset-to-door time and mode of transport serve only as proxies for the geographical barrier, and we recommend systematic recording of travel distance in future prospective studies. The age range was narrow by design (57–75 years), which limits our ability to detect age effects. The number of deaths was small (*n* = 10), so the regression estimates - particularly the wide confidence interval for arrhythmia - should be interpreted cautiously and confirmed in larger, multicenter and prospective studies (15). Use of secondary data precluded analysis of some relevant variables (e.g., precise stroke subtype, infarct volume).

## 5 Conclusion

In a large upper-middle-income setting, elderly stroke patients transferred to a national emergency-medicine center routinely arrive far beyond the reperfusion window, yet in-hospital mortality is low and is associated not with transport time but with neurological severity and cardiac arrhythmia - a pattern shaped both by severity-related self-selection and by the fact that only patients able to survive transfer reach the center. Stroke-system planning in such regions should therefore be risk-based - prioritizing severity- and arrhythmia-driven triage, air-ambulance capacity and regionalized routing -alongside, rather than instead of, efforts to reduce delay. This reframing may be more realistic and more impactful for the many health systems that resemble ours more than they resemble the high-income systems in which the time-is-brain paradigm was forged.

## Data Availability

The raw data supporting the conclusions of this article will be made available by the authors, without undue reservation.
